# Multimodal transformer augmented fusion for speech emotion recognition

**DOI:** 10.3389/fnbot.2023.1181598

**Published:** 2023-05-22

**Authors:** Yuanyuan Wang, Yu Gu, Yifei Yin, Yingping Han, He Zhang, Shuang Wang, Chenyu Li, Dou Quan

**Affiliations:** ^1^School of Artificial Intelligence, Xidian University, Xi'an, China; ^2^Guangzhou Huya Technology Co., Ltd., Guangzhou, China; ^3^School of Journalism and Communication, Northwest University, Xi'an, China

**Keywords:** speech emotion recognition, multimodal enhancement, hybrid fusion, modal interaction, transformer

## Abstract

Speech emotion recognition is challenging due to the subjectivity and ambiguity of emotion. In recent years, multimodal methods for speech emotion recognition have achieved promising results. However, due to the heterogeneity of data from different modalities, effectively integrating different modal information remains a difficulty and breakthrough point of the research. Moreover, in view of the limitations of feature-level fusion and decision-level fusion methods, capturing fine-grained modal interactions has often been neglected in previous studies. We propose a method named multimodal transformer augmented fusion that uses a hybrid fusion strategy, combing feature-level fusion and model-level fusion methods, to perform fine-grained information interaction within and between modalities. A Model-fusion module composed of three Cross-Transformer Encoders is proposed to generate multimodal emotional representation for modal guidance and information fusion. Specifically, the multimodal features obtained by feature-level fusion and text features are used to enhance speech features. Our proposed method outperforms existing state-of-the-art approaches on the IEMOCAP and MELD dataset.

## 1. Introduction

Speech emotion recognition (SER) is a branch of affective computing that aims to determine a person's emotional state from their speech (Ayadi et al., 2011). With the increasing demand for human-computer interaction and emotional interaction, research into SER has practical significance and broad application prospects. A SER system can be used in a vehicle system to determine the driver's psychological state and ensure the safe operation of the vehicle in real-time (Wani et al., 2021). In hospitals and other medical facilities, SER system can help doctors analyze the patient's emotions so as to enhance communication between doctors and patients and help doctors carry out disease diagnosis (Tao and Tan, 2005). SER has also been applied in customer service centers, such as train stations to detect the emotional state of customers in real time, which can help customer service personnel provide more efficient and higher-quality services (Schuller, 2018). SER is also used to help children with autistic who may encounter difficulties in identifying and expressing emotions, thereby improving their socioemotional communication skills (Marchi et al., 2012). However, due to the complexity, subjectivity and ambiguity of emotions, it is still a challenge to accurately recognize emotions from speech.

In recent years, the introduction of multimodal methods for SER has attracted the attention of researchers (Sebe et al., 2005). Emotion is a form of multi-channel expression and people generally use multimodal information such as speech, text, and facial expressions to express emotions (Shimojo and Shams, 2001). Moreover, when noise occurs in one modality, the complementary information of different modalities can increase the robustness of the system. SER based on multimodal fusion information is can therefore be expected to outperform SER based on speech only (Wang et al., 2022).

This paper focuses on using the fusion of speech and text modalities to improve the performance of SER. First, the text modality provides the semantic content. The semantic information is rich and direct, but it is easily affected by the speech recognition task so as to contain ambiguity and bias (Wu J. et al., 2021). The speech modality provides information about the tone, speed, and volume of the speech delivery. Its advantage is that it can help one perceive the speaker 's emotions, but it is difficult to obtain semantic information directly from speech. Second, text can be transcribed from speech, and the text features are part of the speech features. Text and speech can complement each other well (Atmaja et al., 2022). In the event of ambiguity and bias in the text, the emotional information based on the speaker in the speech can be used as a reference. If it is difficult to obtain semantic information from the speech, the text can provide supplementary information.

In view of the limitations of feature-level fusion and decision-level fusion methods, capturing comprehensive and fine-grained modal interaction has often been neglected in previous studies. Compared with decision-level and feature-level fusion methods, model-level fusion can better use the advantages of deep neural networks, better integrate the features of different modalities, and obtain more accurate emotional representations. In hybrid fusion, the advantages of the different fusion strategies can be combined to capture more fine-grained information on intra-modal and inter-modal interaction.

Furthermore, inspired by the attention mechanism, researchers have proposed the transformer (Vaswani et al., 2017), which has achieved promising results in the field of natural language processing (NLP). Transformer has an excellent ability in modeling long-term dependencies in sequences. Although the original Transformer was proposed to solve machine translation problems in the field of NLP, researchers are studying its adaptability to the field of speech signal processing. The multi-head self-attention mechanism can learn long-term time dependence; the multi-head cross-attention mechanism can realize the fusion of different modal features from the model level, and generate intermediate multimodal emotional representations from the common semantic feature space, thereby improving the accuracy of SER.

Therefore, we propose a method named multimodal transformer augmented fusion (MTAF) that uses a hybrid fusion strategy, combining feature-level fusion and model-level fusion methods. The feature-level fusion method is used to fuse the speech and text features to obtain multimodal features. Self-Transformer Encoders are then used to model the long-term time dependence of different modal features. A Model-fusion module is proposed to generate multimodal emotional intermediate representations for modal guidance and information fusion by Cross-Transformer Encoders. Specifically, the multimodal features are used to enhance the speech and text features. The enhanced text features are then used to further enhance the speech features. Finally, the enhanced speech features are used for sentiment classification. The superior performance of MTAF over recent state-of-the-art methods is demonstrated through a variety of experiments conducted on the IEMOCAP and MELD dataset.

## 2. Related work

Although multimodal methods have achieved significant success in the field of SER, there are great differences between modalities, both in their relative independence and in their synchronous or asynchronous information interaction. Hence, effectively integrating the information from different modalities remains a difficulty and breakthrough point of the research (Poria et al., 2017a). In the field of multimodal emotion recognition, researchers have mainly sought to determine at what stage the model could perform the fusion of different modal features.

Fusion methods can be divided into feature-level fusion (early fusion), decision-level fusion (late fusion), model-level fusion, and hybrid-level fusion. Feature-level fusion fuses the features of various modalities (such as visual features, text features, audio features) into general feature vectors, and uses the combined features for analysis. Wu et al. proposed a new deep learning architecture Parallel Inception Convolutional Neural Network (PICNN). They performed convolution in parallel to process sEMG signals from six channels and then used the concatenation method to combine features of different scales directly before entering them into the remainder of the common convolutional neural network (Wu J. et al., 2021; Wu et al., 2022). Joshi et al. combined audio, video, and text features by adding them and sent them to Transformer Encoder (Joshi et al., 2022). The advantage of feature-level fusion is that the low-level features of the data are used in the early stage. More information from the original data is used, and the task is completed based on the correlation between multimodal features (Lian et al., 2020). However, the features obtained by this fusion method belong to different modalities and may vary greatly in many respects. The features must therefore be converted to the same format before the fusion process. Moreover, this fusion method lacks information interaction within the modality, and the high-dimensional feature set may be susceptible to data sparsity problems (Lian et al., 2021), making the method prone to modal information redundancy and leading to data overfitting. The advantages of feature-level fusion are therefore limited.

To overcome this limitation, decision-level fusion uses unimodal decision values and fuses them by ensemble learning (Chen and Zhao, 2020), tensor fusion (Zadeh et al., 2017), or multiplication layer fusion (Mittal et al., 2020). In the decision-level or late fusion process, the features of each modality are examined and classified independently, and the results are fused into decision vectors to obtain the final decision. The advantage of decision-level fusion is that, compared with feature-level fusion, the fusion of decisions obtained from various modalities becomes easier, because decisions generated by multiple modalities often have the same data form (Poria et al., 2016). Another advantage of this fusion process is that each modality can use its most appropriate classifier or model to learn its features (Sun et al., 2021). However, due to the use of different classifiers or models in the analysis task, the learning process in the decision-level fusion phase becomes cumbersome and time-consuming. Moreover, this method must solve the problem of the inability to capture more fine-grained modal dynamics, without taking into account the interaction and correlation between different modalities.

Model-level fusion, in contrast, fuses intermediate representations of different modalities by using various models of deep learning, such as Long Short Term Memory (LSTM), attention, and transformer. In model-level fusion, previous work has used kernel-based methods (Nen et al., 2011) to fuse multimodal features, and showing performance improvements. Subsequently, Charles achieved good results by combining the ability of the graph model (Sutton and McCallum, 2010) to compactly model diverse data with the ability of classification methods to make predictions using a large number of input features. Recently, more advanced methods use attention-based neural networks for model-level fusion. Chen and Jin (2016) proposed a multi-modal conditional attention fusion method to accomplish a continuous multimodal emotion prediction task. Their method can use the temporal information of video combined the historical information and the different levels of features of different modalities, and dynamically give different weights to the visual and auditory modalities input by LSTM at each time step. Poria et al. (2017c) introduced an attention-based fusion mechanism called AT-Fusion that uses the attention score of each modality to fuse multimodal features. It amplifies higher-quality and more informative modalities in the fusion process of multimodal classification, and has achieved promising results in emotion recognition. Wang et al. (2021) proposed a multimodal transformer with shared weights for SER. The proposed network shares cross-modal weights in each Transformer layer to learn the correlation between multiple modalities. However, the effect of the model-level fusion method mainly depends on the fusion model used. This method lacks fine-grained interactions within and between modalities, and cannot make full use of the complementary information between modalities.

Hybrid-level fusion (Poria et al., 2017a) is a combination of the first three fusion methods and is therefore more complex. Sebastian and Pierucci (2019) proposed a combination of early and late fusion techniques, using complementary information from speech and text modalities. Wu W. et al. (2021) proposed a dual-branch structure combining time synchronization and time asynchronous features for multimodal emotion recognition. A time synchronous branch (TSB) captures the correlation between each word in each time step and its acoustic implementation, while time asynchronous branch (TAB) integrates sentence embedding from context sentences. Shen et al. (2020) designed a hierarchical representation of audio at the word, phoneme and frame levels to form more emotionally relevant word-level acoustic features. Xu et al. (2020) established a hierarchical granularity and feature model, which helps to capture more subtle clues and obtain a more complete representation from the original acoustic data. The hybrid fusion method varies depending on the combination of the different fusion methods and is the best and most comprehensive fusion method at present. However, although the hybrid-level fusion method combines the advantages of different fusion methods and makes different modalities interact well, it increases the complexity and training difficulty.

Most of the methods above use a single fusion strategy or a single fusion model and lack fine-grained modal interactions. In contrast to the methods above, our proposed method uses a variety of fusion strategies and multi-level fusion models to capture fine-grained intra-modal and inter-modal information interactions, and achieve high recognition accuracy. This paper presents the multimodal transformer augmented fusion (MTAF) method for emotion recognition, focusing on speech and text domains. The novelty of the work lies in the combination of feature-level and model-level fusion methods and the introduction of a Model-fusion module to facilitate fine-grained interactions between and within modalities. We first use feature-level fusion to perform early modal interactions between the speech and text modalities. Then, we construct the three independent models using Self-Cransformer Encoders to capture the intra-modality dynamics. Finally, a Model-fusion module composed of three Cross-Transformer Encoders to perform late modal interactions. By using a joint model, Fine-grained intermodal dynamic interactions are captured for the speech and text modalities.

## 3. Proposed method

**Figure 1:**
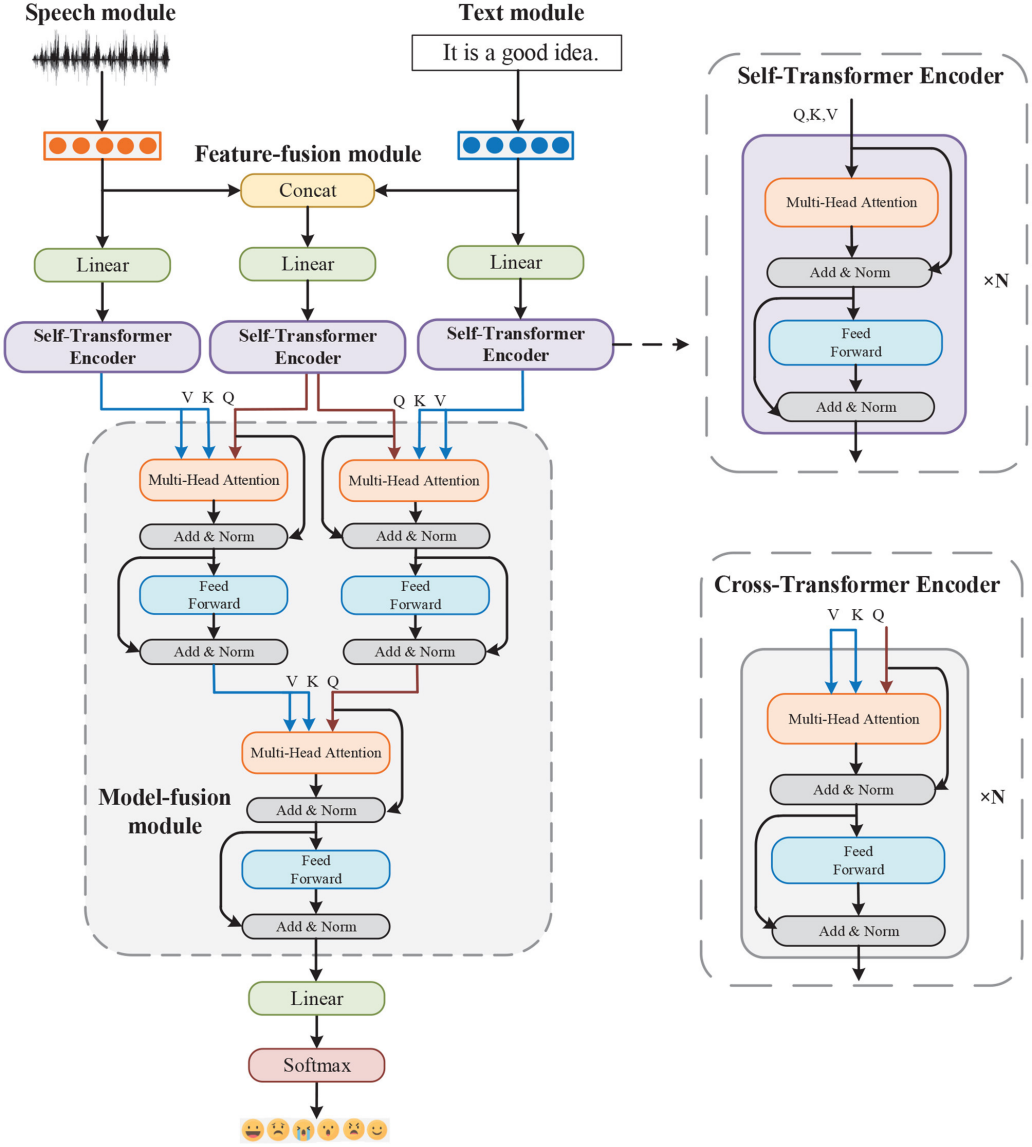
Architecture of the MTAF model.

As shown in Figure 1, we propose a method that uses both speech and text modalities for emotion recognition. The extracted low-level features are fed in sequence to the transformer encoder. The model consists of five parts: Speech, Text, Feature-fusion, Model-fusion modules, and a classification layer.

### 3.1. Feature-level fusion

The input speech feature sequence of an utterance is represented as *x*_*a*_. The text feature sequence of an utterance is represented as *x*_*l*_.

The multimodal feature sequence of an utterance is as follows:


(1) $$x_{e}=\left[x_{a};x_{l}\right]$$

where [ ; ] is the concatenation operator.

### 3.2. Multimodal transformer

We first map the speech, text, and multimodal features obtained in the previous step to the same dimension through a linear layer. The features are then sent to the Self-Transformer Encoder to capture the time dependence. Finally, the Model-fusion module, which is composed of three Cross-Transformer Encoders is used to generate multimodal emotional intermediate representations for modal guidance and information fusion. Specifically, the multimodal features are used to enhance the speech and text features. The enhanced text features are then used to further enhance the speech features.

The core components of the Self-Transformer Encoder and the Cross-Transformer Encoder are a multihead self-attention mechanism and multihead cross-attention mechanism, respectively. Each transformer encoder has *m* layers and *n* attention heads.

#### 3.2.1. Scaled dot-product attention

The query Q, key K, and value V of the multi-head self-attention mechanism come from the same modality. However, for the multi-head cross-attention mechanism, the source modality feature is transformed to the pair of K and V while the target modality feature is transformed into Q. We compute the matrix of outputs as follows:


(2) $$\begin{array}{l} Attention\left(Q,K,V\right)=softmax\left(\frac{QK^{T}}{\sqrt{d_{k}}}\right)V \end{array}$$

where *d*_*k*_ is the dimension of Q.

#### 3.2.2. Multihead attention

Multihead Attention allows the model to focus on information from different presentation subspaces in different locations.


(3) $$\begin{array}{l} Multihead\;\left(Q,K,V\right)=Concat\left({head}_{1},...,{head}_{n}\right)W^{O} \end{array}$$

(4) $$\begin{array}{l} where\;{head}_{i}=Attention\left(QW_{i}^{Q},KW_{i}^{K},VW_{i}^{V}\right) \end{array}$$

where *n* is the number of attention heads; *W*^*O*^, $W_{i}^{Q}$, $W_{i}^{K}$, and $W_{i}^{V}$ are learned model parameters.

### 3.3. Classification layer

After the Model-fusion module, the final multimodal emotional intermediate representation *H* is passed through a fully-connected network and a softmax layer to predict the emotion class with the cross-entropy loss as the cost function:


(5) $$\begin{array}{l} \overset{\sim }{y}=softmax\left(wH+b\right) \end{array}$$

(6) $$\begin{array}{l} Loss=-\frac{1}{N}\;\overset{N}{\underset{i=1}{\sum }}y_{i}log\left(\overset{\sim }{y_{i}}\right)\; \end{array}$$

where *y*_*i*_ is the true label, $\overset{\sim }{y_{i}}$ is the predicted probability distribution from the softmax layer, *w* and *b* are learned model parameters, and *N* is the total number of samples used in training.

## 4. Experiment set-up

### 4.1. Datasets

#### 4.1.1. IEMOCAP

The IEMOCAP (Busso et al., 2008) contains approximately 12 h of audiovisual data. We used the speech and text transcription data which include 7,487 utterances conveying seven emotions: frustration (1,849), neutral (1,708), anger (1,103), sadness (1,084), excitement (1,041), happiness (595), and surprise (107). Excitement is incorporated into happiness. We randomly split the dataset into a training (80%) and a test (20%) set.

#### 4.1.2. MELD

The MELD (Poria et al., 2018) is a new multimodal dataset for emotion recognition. It consists of 13,708 utterances with seven emotions (anger, disgust, fear, joy, neutral, sadness, and surprise) taken from 1,433 dialogues from the classic TV-series Friends. The whole dataset is divided into training, validation, and test sets. In this work, we only use the training and test sets.

### 4.2. Speech and text features

#### 4.2.1. Speech features

Librosa (McFee et al., 2015), a Python package, was used to extract utterance-level speech features. Features with a total of 199 dimensions were extracted, including Mel-Frequency Ceptral Coefficients (MFCC), chroma, pitch, zero-crossing rate, spectral and their statistical measures (HSDs) such as mean, standard deviation, minimum, and maximum.

#### 4.2.2. Text features

The transcripts in the IEMOCAP and MELD dataset were used to extract a 1,890-dimensional Term Frequency-Inverse Document Frequency (TFIDF) feature vector. TFIDF is a numerical statistic that shows the correlation between a word and a document in a collection or corpus (Sahu, 2019).

### 4.3. Implementation details

Through a linear layer, we obtain 256-dimensional speech, text and multimodal features. We feed them into Self-Transformer Encoder, which has 2 Transformer Encoder layers and 4 multi-head attention heads. Next, three newly generated 256-dimensional vectors are sent to the Model-fusion module, which is composed of three Cross-Transformer Encoders. Q is the first residual part of the Cross-Transformer Encoder to perform deep interactions between modalities. Cross-Transformer Encoder and Self-Transformer Encoder have the same number of layers and attention heads. After the Model-fusion module, a 256-dimensional emotional feature vector is finally obtained for sentiment classification.

The training procedure was implemented using PyTorch on a GTX3090. We used the Adam (Kingma and Ba, 2014) optimizer, setting the learning rate to 0.0001. The batch size was 200. To alleviate overfitting, we used the dropout method with a rate of 0.4. We trained the model for at most 50,000 epochs until the accuracy did not change. Weighted accuracy (WA) and unweighted accuracy (UA) were used as the evaluation metrics.

## 5. Experiment results

### 5.1. Comparison with state-of-the-art approaches

To verify the effectiveness of our proposed method, we compared our MTAF with the following thirteen state-of-the-art approaches, all of which use multiple modalities for emotion recognition. These methods can be divided into four groups according to the fusion level.

(1) Feature-level fusion: (a) Audio + Text_LSTM (Sahu, 2019) directly sends the concatenated features to the bidirectional LSTM network. (b) COGMEN (Joshi et al., 2022) propose COntextualized Graph Neural Network based Multimodal Emotion recognitioN (COGMEN) system that leverages local information (inter/intra dependency between speakers) and global information (context).

(2) Decision-level fusion: (a) In Kumar et al. (2021), the audio and textual features were extracted separately using attention-based Gated Recurrent Unit (GRU) and pre-trained Bidirectional Encoder Representations from Transformers (BERT), respectively. Then they were concatenated and used to predict the final emotion class. (b) In MDNN (Zhou et al., 2018), the proposed framework train raw features by groups in local classifiers to avoid high dimensional. Then high-level features of each local classifiers are concatenated as input of a global classifier. (c) bcLSTM (Poria et al., 2017b) propose a LSTM network that takes as input the sequence of utterances in a video and extracts contextual unimodal and multimodal features by modeling the dependencies.

(3) Model-level fusion: (a) Xu et al. (2019) utilized an attention network to learn the alignment between speech and text. (b) MCSAN (Sun et al., 2021) employed the parallel cross- and self attention modules to explicitly model both inter- and intra-modal interactions of audio and text. (c) CAN (Yoonhyung et al., 2020) applied the attention weights of each modality to the other modality in a crossed way so that the CAN gathers the audio and text information from the same time steps based on each modality. (d) CMA + Raw waveform (Krishna and Patil, 2020) applied Cross-modal attention to the output sequences from the audio encoder and text encoder, which helps in finding the interactive information between the audio and text sequences and thus helps improve the performance. (e) CTNet (Lian et al., 2021) proposed to use the transformer-based structure to model intra-modal and cross-modal interactions among multimodal features.

(4) Hybrid-level fusion: (a) Late Fusion-III (Sebastian and Pierucci, 2019) employed various fusion techniques to provide relevance to intermodality dynamics, while keeping the separate models to capture the intra-modality dynamics. (b) HGFM (Xu et al., 2020) took the output of frame-level structure as the input of utterance-level structure and extract the acoustic features of these two levels respectively for effective and complementary fusion. (c) STSER (Chen and Zhao, 2020) applied a multi-scale fusion strategy, including feature fusion and ensemble learning to improve the overall performance.

All the results are listed in Tables 1, 2. On the IEMOCAP dataset, as we can see, our proposed method achieves 72.31% WA and 75.08% UA. Compared with other state-of-the-art approaches, the WA of our method is 0.61 to 14.41% higher and the UA is 0.08 to 26.38% higher. On the MELD dataset, our proposed method achieves 48.12% WA. A 5.82 to 14.12% improvement on other approaches. Although our method is superior to other algorithms, the overall performance on the MELD dataset is not ideal. We speculate that this is because, compared with the IEMOCAP dataset, the MELD dataset should be a relatively large dataset in the field of emotion recognition. Its data comes from the TV program Friends, which is not closer to real life than the data in IEMOCAP. Moreover, the data collection conditions are not as standardized as those of IEMOCAP. The recognition accuracy for MELD is therefore not as high as IEMOCAP. But we do believe that MELD is a good platform to compare and validate our method, because compared with other models, the experimental results of our method improves a lot although the overall results are low.

**Table 1:** Model performance comparisons on the IEMOCAP dataset.

**Model**	**WA (%)**	**UA (%)**
Audio+Text_LSTM	64.20	—
COGMEN	68.2	—
Kumar et al.	71.70	75.00
Xu et al.	70.40	69.50
MCSAN	61.20	56.00
CAN	57.90	48.70
CMA+Raw waveform	—	72.82
CTNet	—	67.60
Late Fusion-III	61.20	59.30
STSER	71.06	72.05
**MTAF**	**72.31**	**75.08**

The bold values indicate the model with the best results, WA and UA with the highest results, respectively.

**Table 2:** Model performance comparisons on the MELD dataset.

**Model**	**WA (%)**
MDNN	34.00
bcLSTM	39.10
HGFM	42.30
**MTAF**	**48.12**

The bold values indicate the model with the best results, WA with the highest results, respectively.

On the surface it seems there is limited improvement in both WA and UA, compared to Kumar's work. Actually, our experimental results were obtained by averaging the results of 10 experiments. Each experiment is carried out under the same experimental conditions, including datasets, model parameters, training and testing processes. The purpose is to reduce the influence of random errors and increase the reliability and stability of the results. The highest experimental results of WA was 73.18% and UA was 75.49% on the IEMOCAP dataset. Compared to Kumar's work, our method achieves 1.48% higher WA and 0.49% higher UA. In addition, the training speed of our proposed model is very fast, so that it can process large amounts of data quickly. The memory occupied by the model is also very small, which is achieved by optimizing and streamlining the model to minimize unnecessary computing and storage operations. We therefore believe that the experimental results show the superiority of our method.

Based on the above experimental results, we analyze the performance enhancements of the model. (1) Though a hybrid fusion strategy, our model combines the advantages of feature-level and decision-level fusion methods to better integrate the two modalities of speech and text. It uses the complementary information of the two modalities to better generate emotional representation. The improvement of the accuracy in the experimental results effectively verifies this point. (2) The multimodal Transformer Encoders achieve fine-grained inter-modal and intra-modal interactions between speech and text modalities well. The Model-fusion module composed of three Cross-Transformer Encoders can generate multimodal emotional intermediate representations for modal guidance and information fusion.

### 5.2. Confusion matrix of experiment

**Figure 2:**
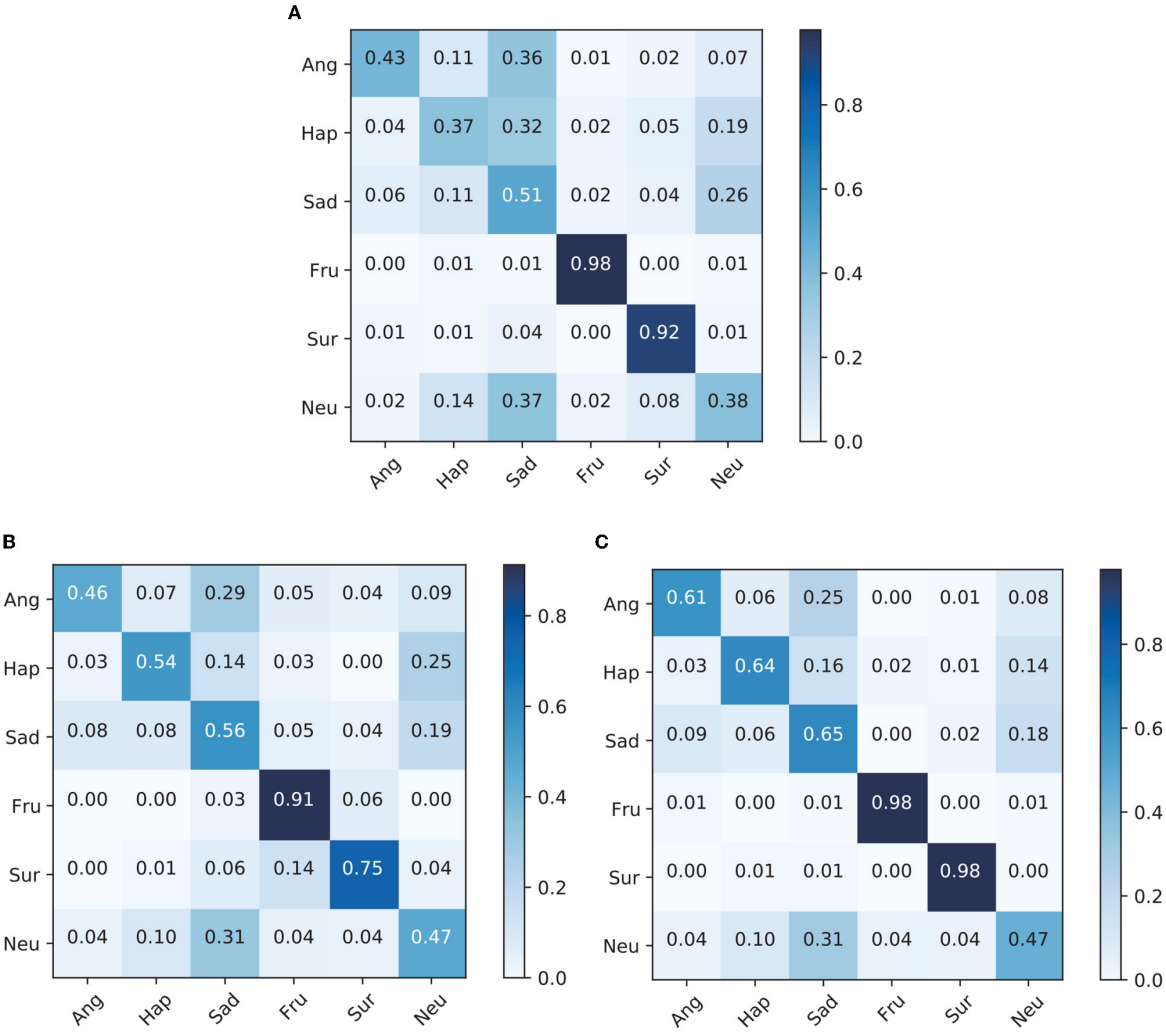
Confusion matrices of each model on the IEMOCAP dataset. **(A)** Speech-only confusion matrix. **(B)** Text-only confusion matrix. **(C)** MTAF confusion matrix.

Figure 2 shows the confusion matrices of models using speech only and text only and the confusion matrix for our proposed model MTAF on the IEMOCAP dataset. The Speech-Only and Text-Only models have a Speech module and Text module, respectively, but not a Model-fusion module.

For the two emotional categories of frustration and neutral from Figure 2, the recognition accuracy of the multimodal model is close to those of the single-modality models. For other emotional categories, the recognition accuracy of the multimodal model is much higher than those of the single-modality models. We also observe a significant improvement of 10–27% recognition accuracy for the happiness category after combining speech and text for emotion recognition. Furthermore, the recognition accuracy of the anger category is significantly improved by 15–18%, and that of the sad category is significantly improved by 9–14%. These experiment results confirm the effectiveness of emotion recognition that combines speech and text modalities. Multimodal methods combine the advantages of different modalities to obtain richer emotional representation. Moreover, complementary information of different modalities can also increase the robustness of the system when noise occurs in one modality.

It is notable that the anger, happiness, and neutral categories are misclassified as sadness with a relatively large probability. Additionally, the happiness and sadness categories are also often classified as neutral. 55% of interpersonal relationships rely on facial expressions or body movements, 38% rely on speech, and only 7% rely on text (Mehrabian, 1971). Facial expressions therefore give very important clues to human emotions (Kim et al., 2017; Dai et al., 2021; Lee et al., 2021). On the basis of speech and text modalities, increasing facial expressions can improve recognition accuracy (Yoon et al., 2019; Kumar et al., 2022). Thus, we infer that humans express these emotions more in facial expressions than in speech and semantic content. These are interesting findings requiring more research and may lead to further improvement in the recognition accuracy.

### 5.3. Ablation study

A variety of ablation experiments were conducted on the IEMOCAP dataset to evaluate the fusion methods, transformer encoders, and model parameters in our proposed method. Tables 3, 4 present the results.

**Table 3:** Ablation study of our proposed method.

**Model**	**WA (%)**	**UA (%)**
Feature fusion	67.15	68.30
Model fusion	63.66	64.95
Without self-transformer encoder	70.09	73.48
Without cross-transformer encoder	69.65	72.24
**MTAF**	**72.31**	**75.08**

The bold values indicate the model with the best results, WA and UA with the highest results, respectively.

**Table 4:** Ablation study of transformer encoder.

***n***	**m**	**WA (%)**	**UA (%)**
1	3	70.89	73.47
**2**	**4**	**72.31**	**75.08**
3	5	71.57	73.86
5	8	70.95	73.64
6	10	70.69	73.17

The bold values indicate the Transformer Encoder layers and multi-head attention heads with the best results, WA and UA with the highest results, respectively.

Table 3 shows the ablation study results for : Feature Fusion, Model Fusion, Without Self-Transformer Encoder, and Without Cross-Transformer Encoder. The Feature Fusion model includes the Feature-fusion module in the middle of Figure 1, but not the Model-fusion module. The Model Fusion model does not include the Feature-fusion module branch and uses only one Cross-Transformer Encoder for multimodal fusion. Then, the Without Self-Transformer Encoder model directly sends extracted low-level speech, text and concatenated multimodal features into the Model-fusion module. Finally, The Without Cross-Transformer Encoder model removes the Model-fusion module and concatenates the outputs of the three branches directly into a fully-connected layer.

In comparison to the Feature Fusion and Model Fusion models, our proposed model, MTAF, achieves 5.16 to 8.65% higher WA and 6.78 to 10.13% higher UA. The experimental results confirm the effectiveness of our proposed hybrid fusion strategy. It combines the advantages of feature-level and model-level fusion methods, captures more fine-grained intra-modal and inter-model interactions, and makes full use of the complementary information of speech and text modalities to obtain richer emotional representation.

When the Self-Transformer Encoder is removed, the model's performance decreases by 2.22% in terms of WA and 1.60% in terms of UA. This finding highlights the importance of using multi-head self-attention mechanisms for intra-modal interaction to capture contextual time dependence. Furthermore, the model's performance decreases when the Cross-Transformer Encoder is removed, which indicates that the multi-head cross-attention mechanisms can integrate features of different modalities for information exchange and generate multimodal emotional intermediate representations.

Table 4 shows the results of using *n* Transformer Encoder layers and *m* multi-head attention heads. Through comparison of the results, we found that *n* = 2, and *m* = 4 achieves the best results. This finding shows that deep-level models are not suitable for SER tasks.

## 6. Conclusion

We propose a method named multimodal transformer augmented fusion that uses a hybrid fusion of both speech and text features, combining feature-level fusion and model-level fusion methods, to effectively integrate different modal information. A Model-fusion module composed of three Cross-Transformer Encoders is proposed to generate multimodal emotional representation for modal guidance and information fusion. Specifically, the Transformer Encoders are used to perform fine-grained dynamic intra- and inter-modality interactions. Moreover, experimental results demonstrate the effectiveness of our proposed method on the IEMOCAP and MELD dataset. In future work, we will try to add facial expressions for multimodal emotion recognition and further improve the accuracy of speech emotion recognition.

## Data availability statement

The original contributions presented in the study are included in the article/supplementary material, further inquiries can be directed to the corresponding author.

## Author contributions

YW: conceptualization and methodology. YG: validation and supervision. All authors contributed to the article and approved the submitted version.

## References

[B1] Atmaja B. T. Sasou A. Akagi M. (2022). Survey on bimodal speech emotion recognition from acoustic and linguistic information fusion. Speech Commun. 140, 11–28. 10.1016/j.specom.2022.03.002

[B2] Ayadi M. E. Kamel M. S. Karray F. (2011). Survey on speech emotion recognition: features, classification schemes, and databases. Pattern Recogn. 44, 572–587. 10.1016/j.patcog.2010.09.020

[B3] Busso C. Bulut M. Lee C.-C. Kazemzadeh A. Mower E. Kim S.. (2008). IEMOCAP: interactive emotional dyadic motion capture database. Lang. Resour. Eval. 42, 335–359. 10.1007/s10579-008-9076-6

[B4] Chen M. Zhao X. (2020). “A multi-scale fusion framework for bimodal speech emotion recognition,” in Interspeech (Cary, NC), 374–378.

[B5] Chen S. Jin Q. (2016). “Multi-modal conditional attention fusion for dimensional emotion prediction,” in Proceedings of the 24th ACM International Conference on Multimedia (New York, NY: ACM), 571–575.

[B6] Dai W. Cahyawijaya S. Liu Z. Fung P. (2021). Multimodal end-to-end sparse model for emotion recognition. arXiv preprint arXiv:2103.09666.

[B7] Joshi A. Bhat A. Jain A. (2022). “Contextualized gnn based multimodal emotion recognition,” in Proceedings of the 2022 Conference of the North American Chapter of the Association for Computational Linguistics: Human Language Technologies (Stroudsburg, PA), 4148–4164.

[B8] Kim H. R. Kim S. J. Lee I. K. (2017). Building emotional machines: Recognizing image emotions through deep neural networks. IEEE Trans. Multimedia. 20, 2980–2992. 10.1109/TMM.2018.2827782

[B9] Kingma D. Ba J. (2014). Adam: a method for stochastic optimization. Comput. Sci.

[B10] Krishna D. N. Patil A. (2020). “Multimodal emotion recognition using cross-modal attention and 1d convolutional neural networks,” in Interspeech (Cary, NC), 4243–4247.

[B11] Kumar P. Kaushik V. Raman B. (2021). “Towards the explainability of multimodal speech emotion recognition,” in InterSpeech (Cary, NC), 1748–1752.

[B12] Kumar P. Malik S. Raman B. (2022). Interpretable multimodal emotion recognition using hybrid fusion of speech and image data. arXiv preprint arXiv:2208.11868.

[B13] Lee S. Han D. K. Ko H. (2021). Multimodal emotion recognition fusion analysis adapting bert with heterogeneous feature unification. IEEE Access. 9, 94557–94572. 10.1109/ACCESS.2021.3092735

[B14] Lian Z. Liu B. Tao J. (2021). “CTNet: conversational transformer network for emotion recognition,” in IEEE/ACM Transactions on Audio, Speech, and Language Processing (New York, NY: IEEE).

[B15] Lian Z. Tao J. Liu B. Huang J. Yang Z. Li R. (2020). “Context-dependent domain adversarial neural network for multimodal emotion recognition,” in Interspeech (Cary, NC), 394–398.

[B16] Marchi E. Schuller B. Batliner A. Fridenzon S. Golan O. (2012). “Emotion in the speech of children with autism spectrum conditions: Prosody and everything else,” in Proceedings 3rd Workshop on Child, Computer and Interaction (WOCCI 2012).

[B17] McFee B. Raffel C. Liang D. Ellis D. P. McVicar M. Battenberg E.. (2015). “librosa: Audio and music signal analysis in Python,” in Proceedings of the 14th Python in Science Conference, 18–25.36116434

[B18] Mehrabian A. (1971). Silent Messages. Belmont, CA: Wadsworth Publishing Company, Inc.

[B19] Mittal T. Bhattacharya U. Chandra R. Bera A. Manocha D. (2020). “M3er: multiplicative multimodal emotion recognition using facial, textual, and speech cues,” in Proceedings of the AAAI Conference on Artificial Intelligence (Menlo Park, CA: AAAI Press), 1359–1367.

[B20] Nen M. Alpayd Ethem, N. (2011). Multiple kernel learning algorithms. J. Mach. Learn. Res. 12, 2211–2268.

[B21] Poria S. Cambria E. Bajpai R. Hussain A. (2017a). A review of affective computing: from unimodal analysis to multimodal fusion. Inform. Fusion 37, 98–125. 10.1016/j.inffus.2017.02.003

[B22] Poria S. Cambria E. Hazarika D. Majumder N. Zadeh A. Morency L.-P. (2017b). “Context-dependent sentiment analysis in user-generated videos,” in Proceedings of the 55th Annual Meeting of the Association for Computational Linguistics (Stroudsburg), 873–883.

[B23] Poria S. Cambria E. Hazarika D. Mazumder N. Zadeh A. Morency L.-P. (2017c). “Multi-level multiple attentions for contextual multimodal sentiment analysis,” in 2017 IEEE International Conference on Data Mining (ICDM) (Piscataway, NJ: IEEE), 1033–1038.

[B24] Poria S. Cambria E. Howard N. Huang G. Hussain A. (2016). Fusing audio, visual and textual clues for sentiment analysis from multimodal content. Neurocomputing 174, 50–59. 10.1016/j.neucom.2015.01.095

[B25] Poria S. Hazarika D. Majumder N. Naik G. Cambria E. Mihalcea R. (2018). “MELD: a multimodal multi-party dataset for emotion recognition in conversations,” in Proceedings of the 57th Annual Meeting of the Association for Computational Linguistics (Stroudsburg). 10.18653/v1/n18-1193 PMC7098709 32219222

[B26] Sahu G. (2019). Multimodal speech emotion recognition and ambiguity resolution. arXiv preprint arXiv:1904.06022.

[B27] Schuller B. W. (2018). Speech emotion recognition two decades in a nutshell, benchmarks, and ongoing trends. Commun. ACM 61, 90–99. 10.1145/3129340

[B28] Sebastian J. Pierucci P. (2019). “Fusion techniques for utterance-level emotion recognition combining speech and transcripts,” in Interspeech (Cary, NC), 51–55.

[B29] Sebe N. Cohen I. Gevers T. Huang T. S. (2005). “Multimodal approaches for emotion recognition: a survey,” in Proceedings of SPIE - The International Society for Optical Engineering (Bellingham).

[B30] Shen G. Lai R. Chen R. Zhang Y. Zhang K. Han Q.. (2020). “Wise: word-level interaction-based multimodal fusion for speech emotion recognition,” in Interspeech (Cary, NC), 369–373.

[B31] Shimojo S. Shams L. (2001). Sensory modalities are not separate modalities: plasticity and interactions. Curr. Opin. Neurobiol. 11, 505–509. 10.1016/S0959-4388(00)00241-5 11502399

[B32] Sun L. Liu B. Tao J. Lian Z. (2021). “Multimodal cross-and self-attention network for speech emotion recognition,” in ICASSP 2021-2021 IEEE International Conference on Acoustics, Speech and Signal Processing (ICASSP) (IEEE), 4275–4279.

[B33] Sutton C. McCallum A. (2010). An introduction to conditional random fields. Found. Trends Mach. Learn. 4, 267–373. 10.1561/2200000013

[B34] Tao J. Tan T. (2005). “Affective computing: a review,” in Affective Computing and Intelligent Interaction: First International Conference, ACII 2005 (Beijing), 981–995.

[B35] Vaswani A. Shazeer N. Parmar N. Uszkoreit J. Jones L. Gomez A. N.. (2017). Attention is all you need. arXiv preprint arXiv:1706.03762.

[B36] Wang C. Ren Y. Zhang N. Cui F. Luo S. (2022). Speech emotion recognition based on multi-feature and multi-lingual fusion. Multimedia Tools Appl. 81, 4897–4907. 10.1007/s11042-021-10553-4

[B37] Wang Y. Shen G. Xu Y. Li J. Zhao Z. (2021). “Learning mutual correlation in multimodal transformer for speech emotion recognition,” in Interspeech (Cary, NC), 4518–4522.

[B38] Wani T. M. Gunawan T. S. Qadri S. Kartiwi M. Ambikairajah E. (2021). A comprehensive review of speech emotion recognition systems. IEEE Access. 9, 47795–47814. 10.1109/ACCESS.2021.3068045

[B39] Wu J. Zhang Y. Xie L. Yan Y. Zhang X. Liu S.. (2022). A novel silent speech recognition approach based on parallel inception convolutional neural network and mel frequency spectral coefficient. Front. Neurorobot. 16, 971446. 10.3389/fnbot.2022.971446 36119717 PMC9478652

[B40] Wu J. Zhao T. Zhang Y. Xie L. Yan Y. Yin E. (2021). “Parallel-inception cnn approach for facial semg based silent speech recognition,” in 2021 43rd Annual International Conference of the IEEE Engineering in Medicine & Biology Society (EMBC), 554–557. 34891354 10.1109/EMBC46164.2021.9630373

[B41] Wu W. Zhang C. Woodland P. C. (2021). “Emotion recognition by fusing time synchronous and time asynchronous representations,” in ICASSP 2021-2021 IEEE International Conference on Acoustics, Speech and Signal Processing (ICASSP) (Piscataway, NJ: IEEE), 6269–6273.

[B42] Xu H. Zhang H. Han K. Wang Y. Peng Y. Li X. (2019). Learning alignment for multimodal emotion recognition from speech. arXiv preprint arXiv:1909.05645.

[B43] Xu Y. Xu H. Zou J. (2020). “HGFM: a hierarchical grained and feature model for acoustic emotion recognition,” in ICASSP 2020-2020 IEEE International Conference on Acoustics, Speech and Signal Processing (ICASSP) (Piscataway, NJ: IEEE), 6499–6503.

[B44] Yoon S. Dey S. Lee H. Jung K. (2019). Attentive modality hopping mechanism for speech emotion recognition. arXiv preprint arXiv:1912.00846.

[B45] Yoonhyung L. Yoon S. Jung K. (2020). “Multimodal speech emotion recognition using cross attention with aligned audio and text,” in Interspeech (Cary, NC), 2717–2721.

[B46] Zadeh A. Chen M. Poria S. Cambria E. Morency L. P. (2017). “Tensor fusion network for multimodal sentiment analysis,” in Proceedings of the 2017 Conference on Empirical Methods in Natural Language Processing (Stroudsburg), 1103–1114.

[B47] Zhou S. Jia J. Wang Q. Dong Y. Yin Y. Lei K. (2018). “Inferring emotion from conversational voice data: a semi-supervised multi-path generative neural network approach,” in Proceedings of the AAAI Conference on Artificial Intelligence (Menlo Park, CA: AAAI Press).

